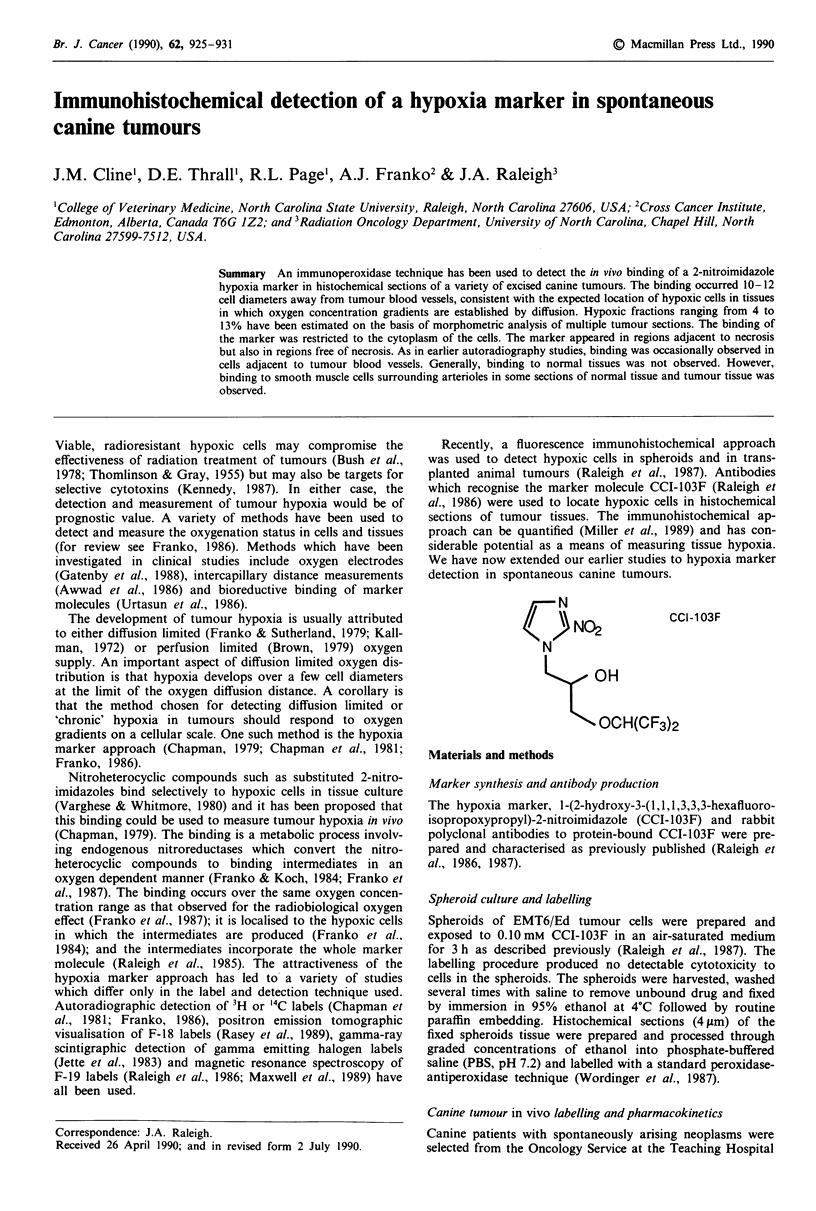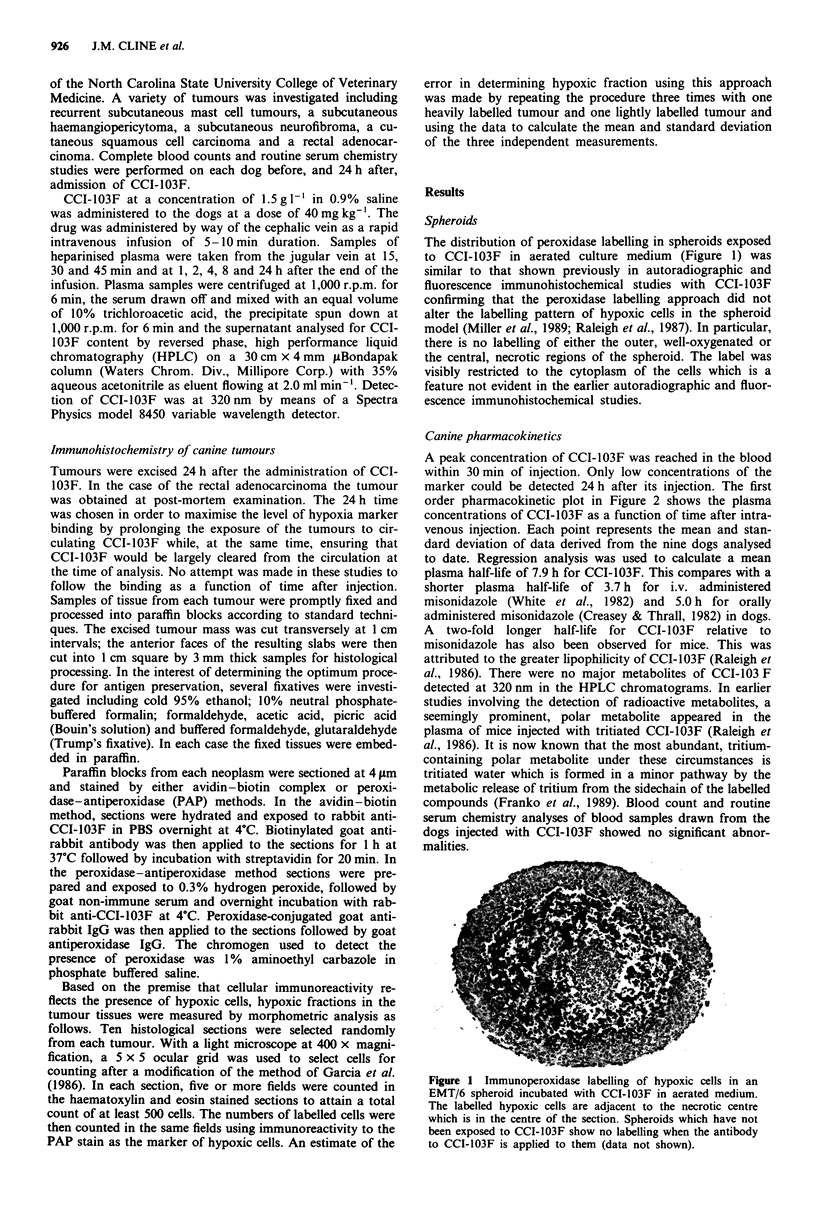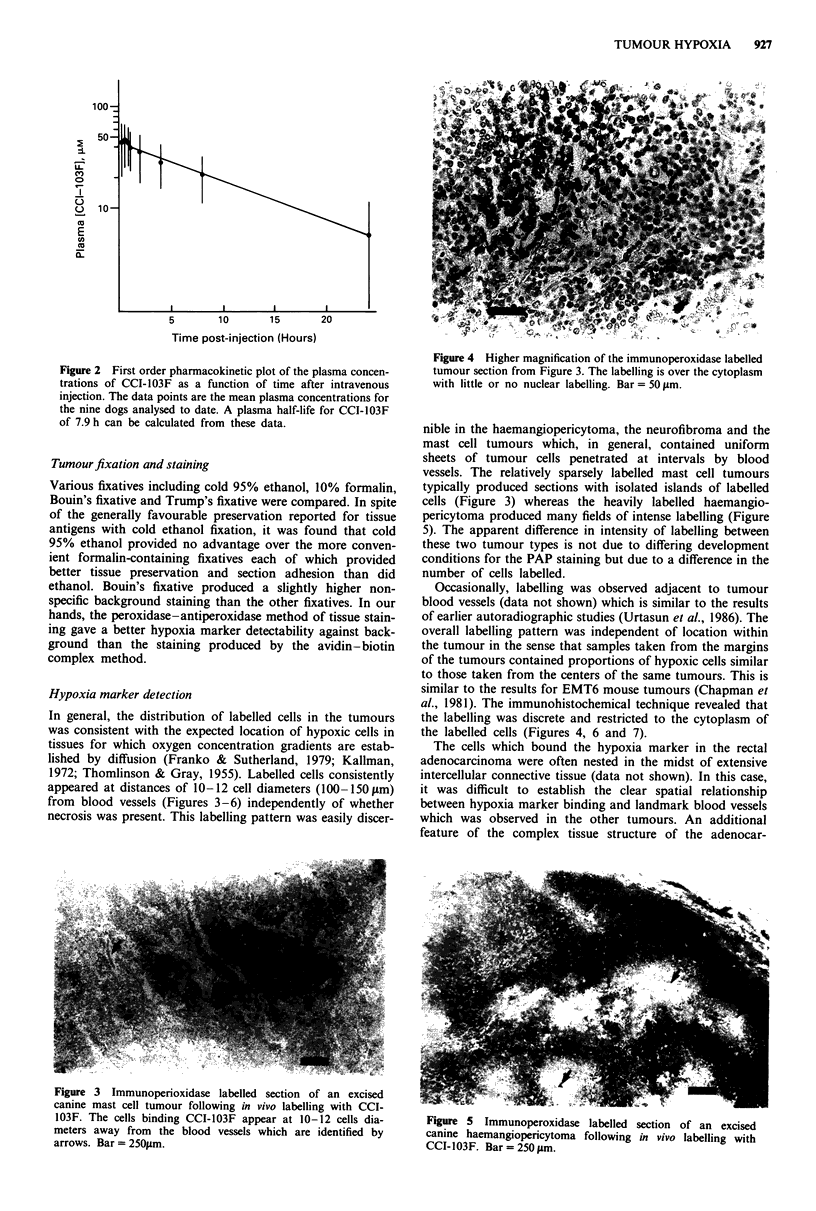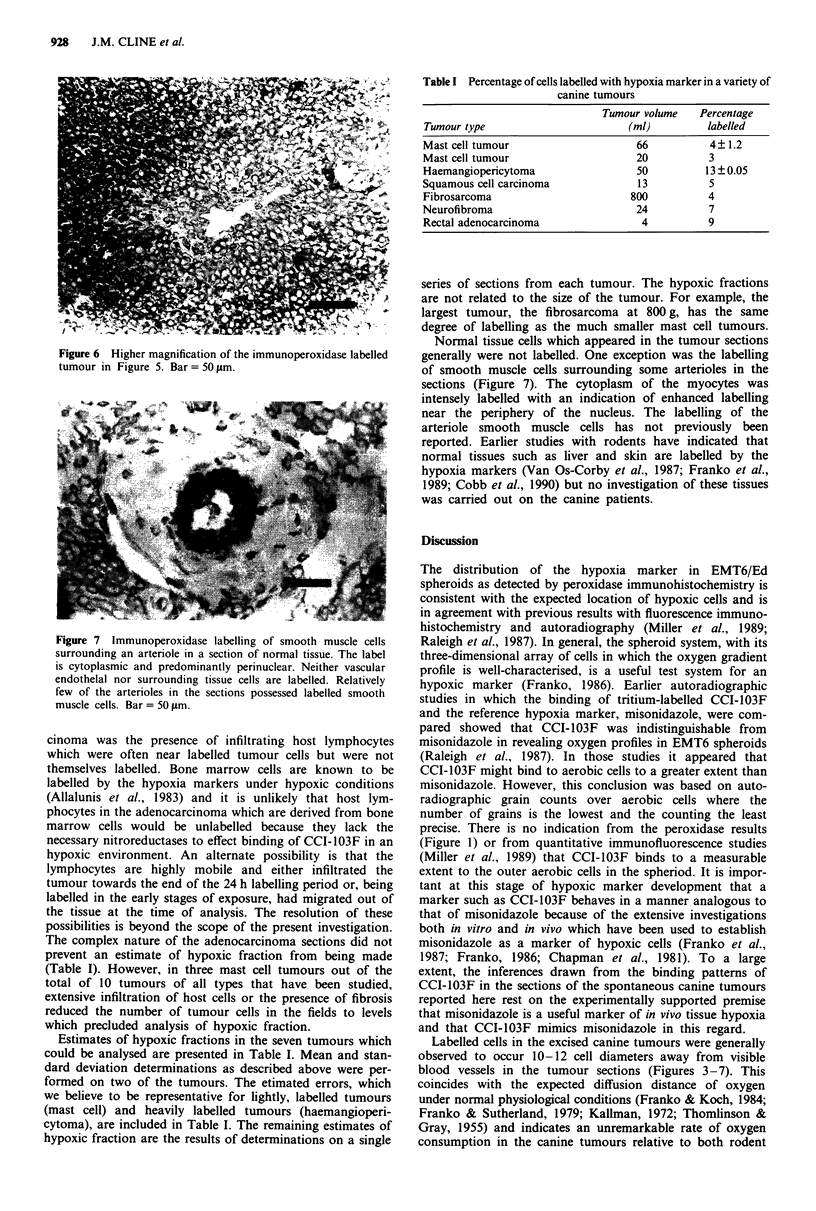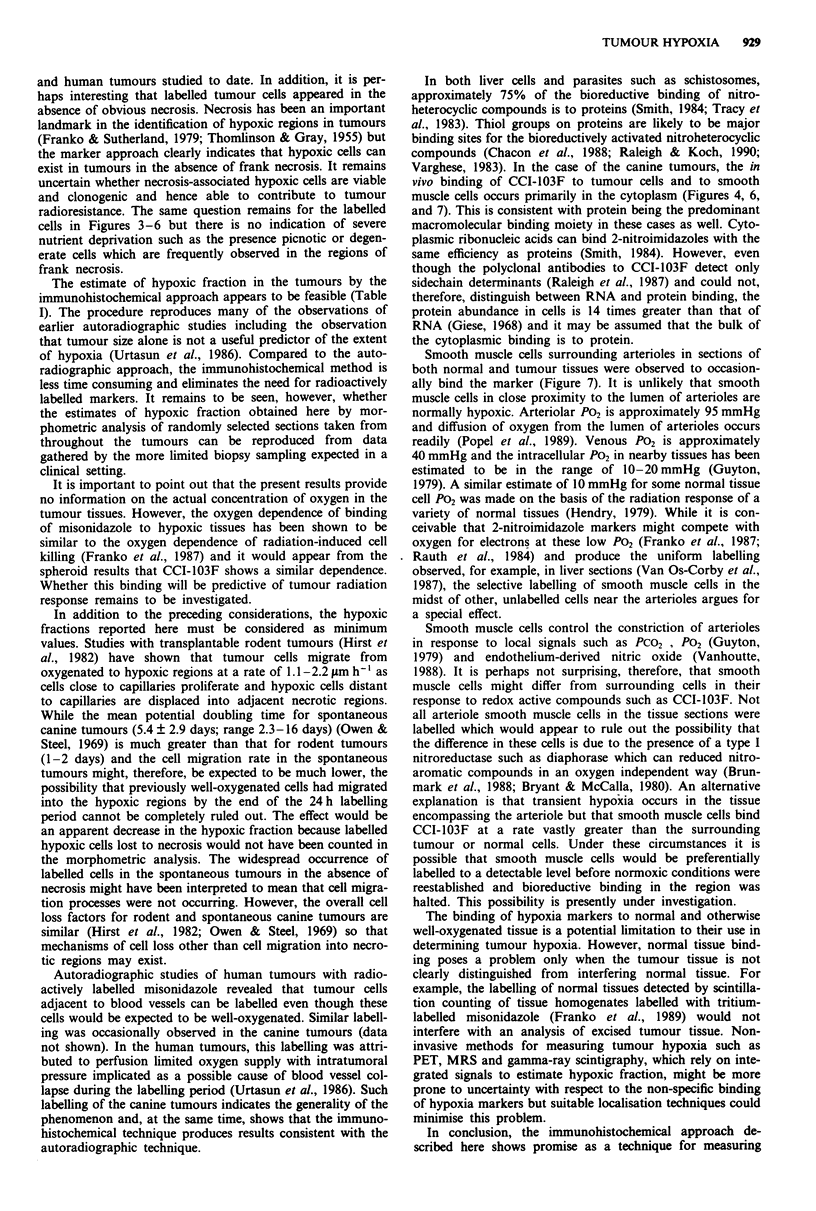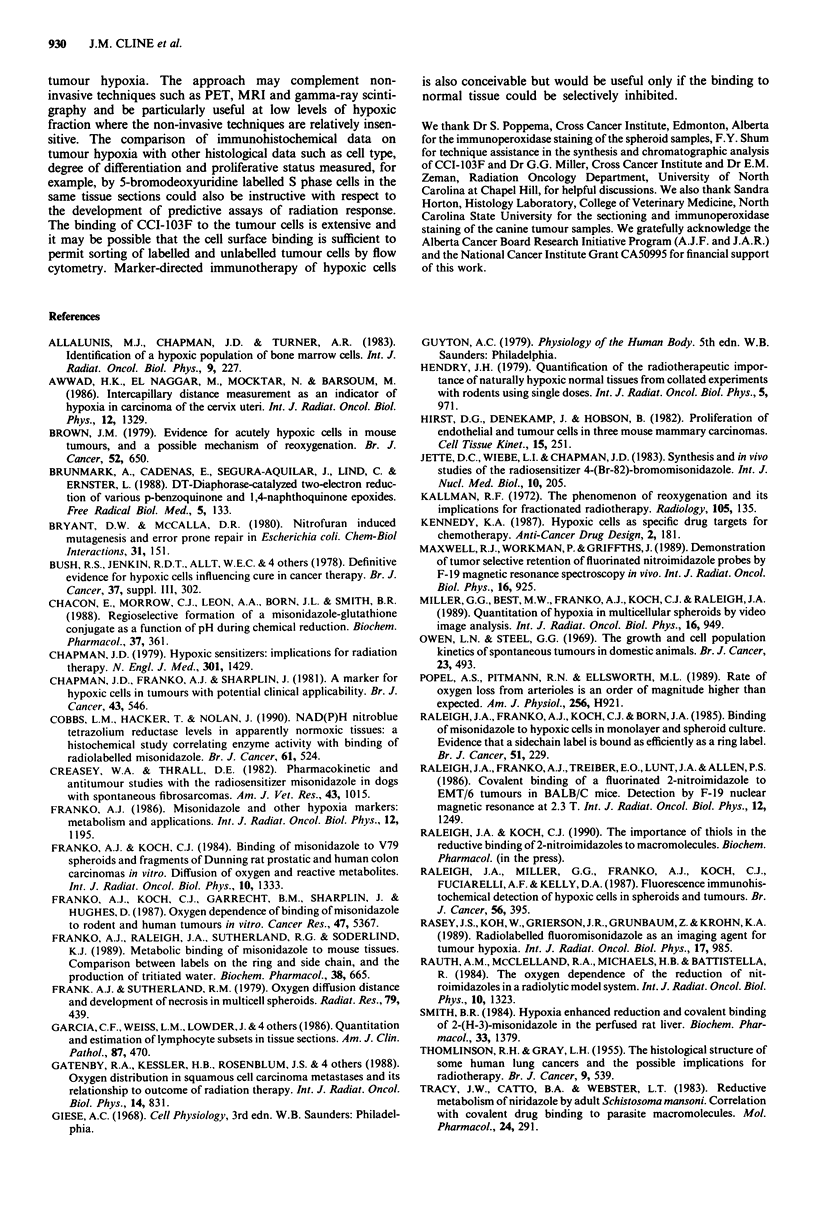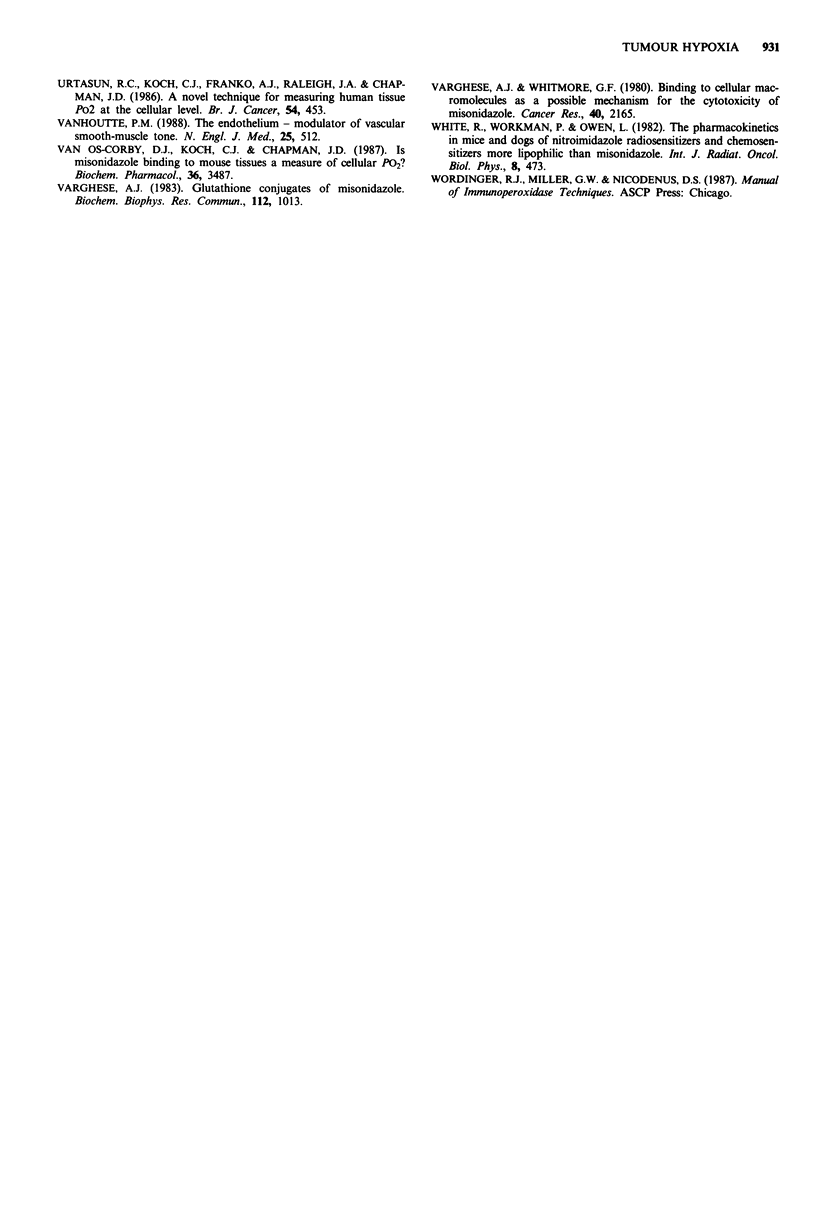# Immunohistochemical detection of a hypoxia marker in spontaneous canine tumours.

**DOI:** 10.1038/bjc.1990.411

**Published:** 1990-12

**Authors:** J. M. Cline, D. E. Thrall, R. L. Page, A. J. Franko, J. A. Raleigh

**Affiliations:** College of Veterinary Medicine, North Carolina State University, Raleigh 27606.

## Abstract

**Images:**


*Br. J. Cancer* (1990), **62**, 925-931 © Macmillan Press Ltd., 1990

### Immunohistochemical detection of a hypoxia marker in spontaneous canine tumours

J.M. Cline[1], D.E. Thrall[1], R.L. Page[1], A.J. Franko[2] & J.A. Raleigh[3]

[1]*College of Veterinary Medicine, North Carolina State University, Raleigh, North Carolina 27606, USA;* [2]*Cross Cancer Institute, Edmonton, Alberta, Canada T6G 1Z2; and* [3]*Radiation Oncology Department, University of North Carolina, Chapel Hill, North Carolina 27599-7512, USA.*

**Summary** An immunoperoxidase technique has been used to detect the *in vivo* binding of a 2-nitroimidazole hypoxia marker in histochemical sections of a variety of excised canine tumours. The binding occurred 10-12 cell diameters away from tumour blood vessels, consistent with the expected location of hypoxic cells in tissues in which oxygen concentration gradients are established by diffusion. Hypoxic fractions ranging from 4 to 13% have been estimated on the basis of morphometric analysis of multiple tumour sections. The binding of the marker was restricted to the cytoplasm of the cells. The marker appeared in regions adjacent to necrosis but also in regions free of necrosis. As in earlier autoradiography studies, binding was occasionally observed in cells adjacent to tumour blood vessels. Generally, binding to normal tissues was not observed. However, binding to smooth muscle cells surrounding arterioles in some sections of normal tissue and tumour tissue was observed.

Viable, radioresistant hypoxic cells may compromise the effectiveness of radiation treatment of tumours (Bush *et al.*, 1978; Thomlinson & Gray, 1955) but may also be targets for selective cytotoxins (Kennedy, 1987). In either case, the detection and measurement of tumour hypoxia would be of prognostic value. A variety of methods have been used to detect and measure the oxygenation status in cells and tissues (for review see Franko, 1986). Methods which have been investigated in clinical studies include oxygen electrodes (Gatenby *et al.*, 1988), intercapillary distance measurements (Awwad *et al.*, 1986) and bioreductive binding of marker molecules (Urtasun *et al.*, 1986).

The development of tumour hypoxia is usually attributed to either diffusion limited (Franko & Sutherland, 1979; Kallman, 1972) or perfusion limited (Brown, 1979) oxygen supply. An important aspect of diffusion limited oxygen distribution is that hypoxia develops over a few cell diameters at the limit of the oxygen diffusion distance. A corollary is that the method chosen for detecting diffusion limited or 'chronic' hypoxia in tumours should respond to oxygen gradients on a cellular scale. One such method is the hypoxia marker approach (Chapman, 1979; Chapman *et al.*, 1981; Franko, 1986).

Nitroheterocyclic compounds such as substituted 2-nitroimidazoles bind selectively to hypoxic cells in tissue culture (Varghese & Whitmore, 1980) and it has been proposed that this binding could be used to measure tumour hypoxia *in vivo* (Chapman, 1979). The binding is a metabolic process involving endogenous nitroreductases which convert the nitroheterocyclic compounds to binding intermediates in an oxygen dependent manner (Franko & Koch, 1984; Franko *et al.*, 1987). The binding occurs over the same oxygen concentration range as that observed for the radiobiological oxygen effect (Franko *et al.*, 1987); it is localised to the hypoxic cells in which the intermediates are produced (Franko *et al.*, 1984); and the intermediates incorporate the whole marker molecule (Raleigh *et al.*, 1985). The attractiveness of the hypoxia marker approach has led to a variety of studies which differ only in the label and detection technique used. Autoradiographic detection of $^{3}H$ or $^{14}C$ labels (Chapman *et al.*, 1981; Franko, 1986), positron emission tomographic visualisation of F-18 labels (Rasey *et al.*, 1989), gamma-ray scintigraphic detection of gamma emitting halogen labels (Jette *et al.*, 1983) and magnetic resonance spectroscopy of F-19 labels (Raleigh *et al.*, 1986; Maxwell *et al.*, 1989) have all been used.

Recently, a fluorescence immunohistochemical approach was used to detect hypoxic cells in spheroids and in transplanted animal tumours (Raleigh *et al.*, 1987). Antibodies which recognise the marker molecule CCI-103F (Raleigh *et al.*, 1986) were used to locate hypoxic cells in histochemical sections of tumour tissues. The immunohistochemical approach can be quantified (Miller *et al.*, 1989) and has considerable potential as a means of measuring tissue hypoxia. We have now extended our earlier studies to hypoxia marker detection in spontaneous canine tumours.

CCI-103F

### Materials and methods

### *Marker synthesis and antibody production*

The hypoxia marker, 1-(2-hydroxy-3-(1,1,1,3,3,3-hexafluoroisopropoxypropyl)-2-nitroimidazole (CCI-103F) and rabbit polyclonal antibodies to protein-bound CCI-103F were prepared and characterised as previously published (Raleigh *et al.*, 1986, 1987).

### *Spheroid culture and labelling*

Spheroids of EMT6/Ed tumour cells were prepared and exposed to 0.10 mM CCI-103F in an air-saturated medium for 3 h as described previously (Raleigh *et al.*, 1987). The labelling procedure produced no detectable cytotoxicity to cells in the spheroids. The spheroids were harvested, washed several times with saline to remove unbound drug and fixed by immersion in 95% ethanol at 4°C followed by routine paraffin embedding. Histochemical sections (4 μm) of the fixed spheroids tissue were prepared and processed through graded concentrations of ethanol into phosphate-buffered saline (PBS, pH 7.2) and labelled with a standard peroxidase-antiperoxidase technique (Wordinger *et al.*, 1987).

### *Canine tumour* in vivo *labelling and pharmacokinetics*

Canine patients with spontaneously arising neoplasms were selected from the Oncology Service at the Teaching Hospital

Correspondence: J.A. Raleigh.
Received 26 April 1990; and in revised form 2 July 1990.

of the North Carolina State University College of Veterinary Medicine. A variety of tumours was investigated including recurrent subcutaneous mast cell tumours, a subcutaneous haemangiopericytoma, a subcutaneous neurofibroma, a cutaneous squamous cell carcinoma and a rectal adenocarcinoma. Complete blood counts and routine serum chemistry studies were performed on each dog before, and 24 h after, admission of CCI-103F.

CCI-103F at a concentration of $1.5\,g\,l^{-1}$ in 0.9% saline was administered to the dogs at a dose of $40\,mg\,kg^{-1}$. The drug was administered by way of the cephalic vein as a rapid intravenous infusion of 5–10 min duration. Samples of heparinised plasma were taken from the jugular vein at 15, 30 and 45 min and at 1, 2, 4, 8 and 24 h after the end of the infusion. Plasma samples were centrifuged at 1,000 r.p.m. for 6 min, the serum drawn off and mixed with an equal volume of 10% trichloroacetic acid, the precipitate spun down at 1,000 r.p.m. for 6 min and the supernatant analysed for CCI-103F content by reversed phase, high performance liquid chromatography (HPLC) on a 30 cm × 4 mm μBondapak column (Waters Chrom. Div., Millipore Corp.) with 35% aqueous acetonitrile as eluent flowing at $2.0\,ml\,min^{-1}$. Detection of CCI-103F was at 320 nm by means of a Spectra Physics model 8450 variable wavelength detector.

*Immunohistochemistry of canine tumours*

Tumours were excised 24 h after the administration of CCI-103F. In the case of the rectal adenocarcinoma the tumour was obtained at post-mortem examination. The 24 h time was chosen in order to maximise the level of hypoxia marker binding by prolonging the exposure of the tumours to circulating CCI-103F while, at the same time, ensuring that CCI-103F would be largely cleared from the circulation at the time of analysis. No attempt was made in these studies to follow the binding as a function of time after injection. Samples of tissue from each tumour were promptly fixed and processed into paraffin blocks according to standard techniques. The excised tumour mass was cut transversely at 1 cm intervals; the anterior faces of the resulting slabs were then cut into 1 cm square by 3 mm thick samples for histological processing. In the interest of determining the optimum procedure for antigen preservation, several fixatives were investigated including cold 95% ethanol; 10% neutral phosphate-buffered formalin; formaldehyde, acetic acid, picric acid (Bouin's solution) and buffered formaldehyde, glutaraldehyde (Trump's fixative). In each case the fixed tissues were embedded in paraffin.

Paraffin blocks from each neoplasm were sectioned at 4 μm and stained by either avidin–biotin complex or peroxidase–antiperoxidase (PAP) methods. In the avidin–biotin method, sections were hydrated and exposed to rabbit anti-CCI-103F in PBS overnight at 4°C. Biotinylated goat anti-rabbit antibody was then applied to the sections for 1 h at 37°C followed by incubation with streptavidin for 20 min. In the peroxidase–antiperoxidase method sections were prepared and exposed to 0.3% hydrogen peroxide, followed by goat non-immune serum and overnight incubation with rabbit anti-CCI-103F at 4°C. Peroxidase-conjugated goat anti-rabbit IgG was then applied to the sections followed by goat antiperoxidase IgG. The chromogen used to detect the presence of peroxidase was 1% aminoethyl carbazole in phosphate buffered saline.

Based on the premise that cellular immunoreactivity reflects the presence of hypoxic cells, hypoxic fractions in the tumour tissues were measured by morphometric analysis as follows. Ten histological sections were selected randomly from each tumour. With a light microscope at 400 × magnification, a 5 × 5 ocular grid was used to select cells for counting after a modification of the method of Garcia et al. (1986). In each section, five or more fields were counted in the haematoxylin and eosin stained sections to attain a total count of at least 500 cells. The numbers of labelled cells were then counted in the same fields using immunoreactivity to the PAP stain as the marker of hypoxic cells. An estimate of the error in determining hypoxic fraction using this approach was made by repeating the procedure three times with one heavily labelled tumour and one lightly labelled tumour and using the data to calculate the mean and standard deviation of the three independent measurements.

### Results

*Spheroids*

The distribution of peroxidase labelling in spheroids exposed to CCI-103F in aerated culture medium (Figure 1) was similar to that shown previously in autoradiographic and fluorescence immunohistochemical studies with CCI-103F confirming that the peroxidase labelling approach did not alter the labelling pattern of hypoxic cells in the spheroid model (Miller et al., 1989; Raleigh et al., 1987). In particular, there is no labelling of either the outer, well-oxygenated or the central, necrotic regions of the spheroid. The label was visibly restricted to the cytoplasm of the cells which is a feature not evident in the earlier autoradiographic and fluorescence immunohistochemical studies.

*Canine pharmacokinetics*

A peak concentration of CCI-103F was reached in the blood within 30 min of injection. Only low concentrations of the marker could be detected 24 h after its injection. The first order pharmacokinetic plot in Figure 2 shows the plasma concentrations of CCI-103F as a function of time after intravenous injection. Each point represents the mean and standard deviation of data derived from the nine dogs analysed to date. Regression analysis was used to calculate a mean plasma half-life of 7.9 h for CCI-103F. This compares with a shorter plasma half-life of 3.7 h for i.v. administered misonidazole (White et al., 1982) and 5.0 h for orally administered misonidazole (Creasey & Thrall, 1982) in dogs. A two-fold longer half-life for CCI-103F relative to misonidazole has also been observed for mice. This was attributed to the greater lipophilicity of CCI-103F (Raleigh et al., 1986). There were no major metabolites of CCI-103 F detected at 320 nm in the HPLC chromatograms. In earlier studies involving the detection of radioactive metabolites, a seemingly prominent, polar metabolite appeared in the plasma of mice injected with tritiated CCI-103F (Raleigh et al., 1986). It is now known that the most abundant, tritium-containing polar metabolite under these circumstances is tritiated water which is formed in a minor pathway by the metabolic release of tritium from the sidechain of the labelled compounds (Franko et al., 1989). Blood count and routine serum chemistry analyses of blood samples drawn from the dogs injected with CCI-103F showed no significant abnormalities.

**Figure 1** Immunoperoxidase labelling of hypoxic cells in an EMT/6 spheroid incubated with CCI-103F in aerated medium. The labelled hypoxic cells are adjacent to the necrotic centre which is in the centre of the section. Spheroids which have not been exposed to CCI-103F show no labelling when the antibody to CCI-103F is applied to them (data not shown).

**Figure 2** First order pharmacokinetic plot of the plasma concentrations of CCI-103F as a function of time after intravenous injection. The data points are the mean plasma concentrations for the nine dogs analysed to date. A plasma half-life for CCI-103F of 7.9 h can be calculated from these data.

*Tumour fixation and staining*

Various fixatives including cold 95% ethanol, 10% formalin, Bouin's fixative and Trump's fixative were compared. In spite of the generally favourable preservation reported for tissue antigens with cold ethanol fixation, it was found that cold 95% ethanol provided no advantage over the more convenient formalin-containing fixatives each of which provided better tissue preservation and section adhesion than did ethanol. Bouin's fixative produced a slightly higher non-specific background staining than the other fixatives. In our hands, the peroxidase-antiperoxidase method of tissue staining gave a better hypoxia marker detectability against background than the staining produced by the avidin-biotin complex method.

*Hypoxia marker detection*

In general, the distribution of labelled cells in the tumours was consistent with the expected location of hypoxic cells in tissues for which oxygen concentration gradients are established by diffusion (Franko & Sutherland, 1979; Kallman, 1972; Thomlinson & Gray, 1955). Labelled cells consistently appeared at distances of 10–12 cell diameters (100–150 μm) from blood vessels (Figures 3–6) independently of whether necrosis was present. This labelling pattern was easily discer-

**Figure 4** Higher magnification of the immunoperoxidase labelled tumour section from Figure 3. The labelling is over the cytoplasm with little or no nuclear labelling. Bar = 50 μm.

nible in the haemangiopericytoma, the neurofibroma and the mast cell tumours which, in general, contained uniform sheets of tumour cells penetrated at intervals by blood vessels. The relatively sparsely labelled mast cell tumours typically produced sections with isolated islands of labelled cells (Figure 3) whereas the heavily labelled haemangiopericytoma produced many fields of intense labelling (Figure 5). The apparent difference in intensity of labelling between these two tumour types is not due to differing development conditions for the PAP staining but due to a difference in the number of cells labelled.

Occasionally, labelling was observed adjacent to tumour blood vessels (data not shown) which is similar to the results of earlier autoradiographic studies (Urtasun *et al.*, 1986). The overall labelling pattern was independent of location within the tumour in the sense that samples taken from the margins of the tumours contained proportions of hypoxic cells similar to those taken from the centers of the same tumours. This is similar to the results for EMT6 mouse tumours (Chapman *et al.*, 1981). The immunohistochemical technique revealed that the labelling was discrete and restricted to the cytoplasm of the labelled cells (Figures 4, 6 and 7).

The cells which bound the hypoxia marker in the rectal adenocarcinoma were often nested in the midst of extensive intercellular connective tissue (data not shown). In this case, it was difficult to establish the clear spatial relationship between hypoxia marker binding and landmark blood vessels which was observed in the other tumours. An additional feature of the complex tissue structure of the adenocar-

**Figure 3** Immunoperioxidase labelled section of an excised canine mast cell tumour following *in vivo* labelling with CCI-103F. The cells binding CCI-103F appear at 10–12 cells diameters away from the blood vessels which are identified by arrows. Bar = 250μm.

**Figure 5** Immunoperoxidase labelled section of an excised canine haemangiopericytoma following *in vivo* labelling with CCI-103F. Bar = 250 μm.

**Figure 6** Higher magnification of the immunoperoxidase labelled tumour in Figure 5. Bar = 50 μm.

**Figure 7** Immunoperoxidase labelling of smooth muscle cells surrounding an arteriole in a section of normal tissue. The label is cytoplasmic and predominantly perinuclear. Neither vascular endothelal nor surrounding tissue cells are labelled. Relatively few of the arterioles in the sections possessed labelled smooth muscle cells. Bar = 50 μm.

cinoma was the presence of infiltrating host lymphocytes which were often near labelled tumour cells but were not themselves labelled. Bone marrow cells are known to be labelled by the hypoxia markers under hypoxic conditions (Allalunis *et al.*, 1983) and it is unlikely that host lymphocytes in the adenocarcinoma which are derived from bone marrow cells would be unlabelled because they lack the necessary nitroreductases to effect binding of CCI-103F in an hypoxic environment. An alternate possibility is that the lymphocytes are highly mobile and either infiltrated the tumour towards the end of the 24 h labelling period or, being labelled in the early stages of exposure, had migrated out of the tissue at the time of analysis. The resolution of these possibilities is beyond the scope of the present investigation. The complex nature of the adenocarcinoma sections did not prevent an estimate of hypoxic fraction from being made (Table I). However, in three mast cell tumours out of the total of 10 tumours of all types that have been studied, extensive infiltration of host cells or the presence of fibrosis reduced the number of tumour cells in the fields to levels which precluded analysis of hypoxic fraction.

Estimates of hypoxic fractions in the seven tumours which could be analysed are presented in Table I. Mean and standard deviation determinations as described above were performed on two of the tumours. The estimated errors, which we believe to be representative for lightly, labelled tumours (mast cell) and heavily labelled tumours (haemangiopericytoma), are included in Table I. The remaining estimates of hypoxic fraction are the results of determinations on a single

**Table I** Percentage of cells labelled with hypoxia marker in a variety of canine tumours

| *Tumour type* | *Tumour volume (ml)* | *Percentage labelled* |
|---|---|---|
| Mast cell tumour | 66 | 4 ± 1.2 |
| Mast cell tumour | 20 | 3 |
| Haemangiopericytoma | 50 | 13 ± 0.05 |
| Squamous cell carcinoma | 13 | 5 |
| Fibrosarcoma | 800 | 4 |
| Neurofibroma | 24 | 7 |
| Rectal adenocarcinoma | 4 | 9 |

series of sections from each tumour. The hypoxic fractions are not related to the size of the tumour. For example, the largest tumour, the fibrosarcoma at 800 g, has the same degree of labelling as the much smaller mast cell tumours.

Normal tissue cells which appeared in the tumour sections generally were not labelled. One exception was the labelling of smooth muscle cells surrounding some arterioles in the sections (Figure 7). The cytoplasm of the myocytes was intensely labelled with an indication of enhanced labelling near the periphery of the nucleus. The labelling of the arteriole smooth muscle cells has not previously been reported. Earlier studies with rodents have indicated that normal tissues such as liver and skin are labelled by the hypoxia markers (Van Os-Corby *et al.*, 1987; Franko *et al.*, 1989; Cobb *et al.*, 1990) but no investigation of these tissues was carried out on the canine patients.

### Discussion

The distribution of the hypoxia marker in EMT6/Ed spheroids as detected by peroxidase immunohistochemistry is consistent with the expected location of hypoxic cells and is in agreement with previous results with fluorescence immunohistochemistry and autoradiography (Miller *et al.*, 1989; Raleigh *et al.*, 1987). In general, the spheroid system, with its three-dimensional array of cells in which the oxygen gradient profile is well-characterised, is a useful test system for an hypoxic marker (Franko, 1986). Earlier autoradiographic studies in which the binding of tritium-labelled CCI-103F and the reference hypoxia marker, misonidazole, were compared showed that CCI-103F was indistinguishable from misonidazole in revealing oxygen profiles in EMT6 spheroids (Raleigh *et al.*, 1987). In those studies it appeared that CCI-103F might bind to aerobic cells to a greater extent than misonidazole. However, this conclusion was based on autoradiographic grain counts over aerobic cells where the number of grains is the lowest and the counting the least precise. There is no indication from the peroxidase results (Figure 1) or from quantitative immunofluorescence studies (Miller *et al.*, 1989) that CCI-103F binds to a measurable extent to the outer aerobic cells in the spheriod. It is important at this stage of hypoxic marker development that a marker such as CCI-103F behaves in a manner analogous to that of misonidazole because of the extensive investigations both *in vitro* and *in vivo* which have been used to establish misonidazole as a marker of hypoxic cells (Franko *et al.*, 1987; Franko, 1986; Chapman *et al.*, 1981). To a large extent, the inferences drawn from the binding patterns of CCI-103F in the sections of the spontaneous canine tumours reported here rest on the experimentally supported premise that misonidazole is a useful marker of *in vivo* tissue hypoxia and that CCI-103F mimics misonidazole in this regard.

Labelled cells in the excised canine tumours were generally observed to occur 10–12 cell diameters away from visible blood vessels in the tumour sections (Figures 3–7). This coincides with the expected diffusion distance of oxygen under normal physiological conditions (Franko & Koch, 1984; Franko & Sutherland, 1979; Kallman, 1972; Thomlinson & Gray, 1955) and indicates an unremarkable rate of oxygen consumption in the canine tumours relative to both rodent and human tumours studied to date. In addition, it is perhaps interesting that labelled tumour cells appeared in the absence of obvious necrosis. Necrosis has been an important landmark in the identification of hypoxic regions in tumours (Franko & Sutherland, 1979; Thomlinson & Gray, 1955) but the marker approach clearly indicates that hypoxic cells can exist in tumours in the absence of frank necrosis. It remains uncertain whether necrosis-associated hypoxic cells are viable and clonogenic and hence able to contribute to tumour radioresistance. The same question remains for the labelled cells in Figures 3–6 but there is no indication of severe nutrient deprivation such as the presence picnotic or degenerate cells which are frequently observed in the regions of frank necrosis.

The estimate of hypoxic fraction in the tumours by the immunohistochemical approach appears to be feasible (Table I). The procedure reproduces many of the observations of earlier autoradiographic studies including the observation that tumour size alone is not a useful predictor of the extent of hypoxia (Urtasun *et al.*, 1986). Compared to the autoradiographic approach, the immunohistochemical method is less time consuming and eliminates the need for radioactively labelled markers. It remains to be seen, however, whether the estimates of hypoxic fraction obtained here by morphometric analysis of randomly selected sections taken from throughout the tumours can be reproduced from data gathered by the more limited biopsy sampling expected in a clinical setting.

It is important to point out that the present results provide no information on the actual concentration of oxygen in the tumour tissues. However, the oxygen dependence of binding of misonidazole to hypoxic tissues has been shown to be similar to the oxygen dependence of radiation-induced cell killing (Franko *et al.*, 1987) and it would appear from the spheroid results that CCI-103F shows a similar dependence. Whether this binding will be predictive of tumour radiation response remains to be investigated.

In addition to the preceding considerations, the hypoxic fractions reported here must be considered as minimum values. Studies with transplantable rodent tumours (Hirst *et al.*, 1982) have shown that tumour cells migrate from oxygenated to hypoxic regions at a rate of $1.1-2.2\ \mu m\ h^{-1}$ as cells close to capillaries proliferate and hypoxic cells distant to capillaries are displaced into adjacent necrotic regions. While the mean potential doubling time for spontaneous canine tumours ($5.4 \pm 2.9$ days; range 2.3–16 days) (Owen & Steel, 1969) is much greater than that for rodent tumours (1–2 days) and the cell migration rate in the spontaneous tumours might, therefore, be expected to be much lower, the possibility that previously well-oxygenated cells had migrated into the hypoxic regions by the end of the 24 h labelling period cannot be completely ruled out. The effect would be an apparent decrease in the hypoxic fraction because labelled hypoxic cells lost to necrosis would not have been counted in the morphometric analysis. The widespread occurrence of labelled cells in the spontaneous tumours in the absence of necrosis might have been interpreted to mean that cell migration processes were not occurring. However, the overall cell loss factors for rodent and spontaneous canine tumours are similar (Hirst *et al.*, 1982; Owen & Steel, 1969) so that mechanisms of cell loss other than cell migration into necrotic regions may exist.

Autoradiographic studies of human tumours with radioactively labelled misonidazole revealed that tumour cells adjacent to blood vessels can be labelled even though these cells would be expected to be well-oxygenated. Similar labelling was occasionally observed in the canine tumours (data not shown). In the human tumours, this labelling was attributed to perfusion limited oxygen supply with intratumoral pressure implicated as a possible cause of blood vessel collapse during the labelling period (Urtasun *et al.*, 1986). Such labelling of the canine tumours indicates the generality of the phenomenon and, at the same time, shows that the immunohistochemical technique produces results consistent with the autoradiographic technique.

In both liver cells and parasites such as schistosomes, approximately 75% of the bioreductive binding of nitroheterocyclic compounds is to proteins (Smith, 1984; Tracy *et al.*, 1983). Thiol groups on proteins are likely to be major binding sites for the bioreductively activated nitroheterocyclic compounds (Chacon *et al.*, 1988; Raleigh & Koch, 1990; Varghese, 1983). In the case of the canine tumours, the *in vivo* binding of CCI-103F to tumour cells and to smooth muscle cells occurs primarily in the cytoplasm (Figures 4, 6, and 7). This is consistent with protein being the predominant macromolecular binding moiety in these cases as well. Cytoplasmic ribonucleic acids can bind 2-nitroimidazoles with the same efficiency as proteins (Smith, 1984). However, even though the polyclonal antibodies to CCI-103F detect only sidechain determinants (Raleigh *et al.*, 1987) and could not, therefore, distinguish between RNA and protein binding, the protein abundance in cells is 14 times greater than that of RNA (Giese, 1968) and it may be assumed that the bulk of the cytoplasmic binding is to protein.

Smooth muscle cells surrounding arterioles in sections of both normal and tumour tissues were observed to occasionally bind the marker (Figure 7). It is unlikely that smooth muscle cells in close proximity to the lumen of arterioles are normally hypoxic. Arteriolar $Po_2$ is approximately 95 mmHg and diffusion of oxygen from the lumen of arterioles occurs readily (Popel *et al.*, 1989). Venous $Po_2$ is approximately 40 mmHg and the intracellular $Po_2$ in nearby tissues has been estimated to be in the range of 10–20 mmHg (Guyton, 1979). A similar estimate of 10 mmHg for some normal tissue cell $Po_2$ was made on the basis of the radiation response of a variety of normal tissues (Hendry, 1979). While it is conceivable that 2-nitroimidazole markers might compete with oxygen for electrons at these low $Po_2$ (Franko *et al.*, 1987; Rauth *et al.*, 1984) and produce the uniform labelling observed, for example, in liver sections (Van Os-Corby *et al.*, 1987), the selective labelling of smooth muscle cells in the midst of other, unlabelled cells near the arterioles argues for a special effect.

Smooth muscle cells control the constriction of arterioles in response to local signals such as $Pco_2$ , $Po_2$ (Guyton, 1979) and endothelium-derived nitric oxide (Vanhoutte, 1988). It is perhaps not surprising, therefore, that smooth muscle cells might differ from surrounding cells in their response to redox active compounds such as CCI-103F. Not all arteriole smooth muscle cells in the tissue sections were labelled which would appear to rule out the possibility that the difference in these cells is due to the presence of a type I nitroreductase such as diaphorase which can reduced nitroaromatic compounds in an oxygen independent way (Brunmark *et al.*, 1988; Bryant & McCalla, 1980). An alternative explanation is that transient hypoxia occurs in the tissue encompassing the arteriole but that smooth muscle cells bind CCI-103F at a rate vastly greater than the surrounding tumour or normal cells. Under these circumstances it is possible that smooth muscle cells would be preferentially labelled to a detectable level before normoxic conditions were reestablished and bioreductive binding in the region was halted. This possibility is presently under investigation.

The binding of hypoxia markers to normal and otherwise well-oxygenated tissue is a potential limitation to their use in determining tumour hypoxia. However, normal tissue binding poses a problem only when the tumour tissue is not clearly distinguished from interfering normal tissue. For example, the labelling of normal tissues detected by scintillation counting of tissue homogenates labelled with tritium-labelled misonidazole (Franko *et al.*, 1989) would not interfere with an analysis of excised tumour tissue. Non-invasive methods for measuring tumour hypoxia such as PET, MRS and gamma-ray scintigraphy, which rely on integrated signals to estimate hypoxic fraction, might be more prone to uncertainty with respect to the non-specific binding of hypoxia markers but suitable localisation techniques could minimise this problem.

In conclusion, the immunohistochemical approach described here shows promise as a technique for measuring tumour hypoxia. The approach may complement non-invasive techniques such as PET, MRI and gamma-ray scintigraphy and be particularly useful at low levels of hypoxic fraction where the non-invasive techniques are relatively insensitive. The comparison of immunohistochemical data on tumour hypoxia with other histological data such as cell type, degree of differentiation and proliferative status measured, for example, by 5-bromodeoxyuridine labelled S phase cells in the same tissue sections could also be instructive with respect to the development of predictive assays of radiation response. The binding of CCI-103F to the tumour cells is extensive and it may be possible that the cell surface binding is sufficient to permit sorting of labelled and unlabelled tumour cells by flow cytometry. Marker-directed immunotherapy of hypoxic cells is also conceivable but would be useful only if the binding to normal tissue could be selectively inhibited.

We thank Dr S. Poppema, Cross Cancer Institute, Edmonton, Alberta for the immunoperoxidase staining of the spheroid samples, F.Y. Shum for technique assistance in the synthesis and chromatographic analysis of CCI-103F and Dr G.G. Miller, Cross Cancer Institute and Dr E.M. Zeman, Radiation Oncology Department, University of North Carolina at Chapel Hill, for helpful discussions. We also thank Sandra Horton, Histology Laboratory, College of Veterinary Medicine, North Carolina State University for the sectioning and immunoperoxidase staining of the canine tumour samples. We gratefully acknowledge the Alberta Cancer Board Research Initiative Program (A.J.F. and J.A.R.) and the National Cancer Institute Grant CA50995 for financial support of this work.

### References

ALLALUNIS, M.J., CHAPMAN, J.D. & TURNER, A.R. (1983). Identification of a hypoxic population of bone marrow cells. *Int. J. Radiat. Oncol. Biol. Phys.*, **9**, 227.

AWWAD, H.K., EL NAGGAR, M., MOCKTAR, N. & BARSOUM, M. (1986). Intercapillary distance measurement as an indicator of hypoxia in carcinoma of the cervix uteri. *Int. J. Radiat. Oncol. Biol. Phys.*, **12**, 1329.

BROWN, J.M. (1979). Evidence for acutely hypoxic cells in mouse tumours, and a possible mechanism of reoxygenation. *Br. J. Cancer*, **52**, 650.

BRUNMARK, A., CADENAS, E., SEGURA-AQUILAR, J., LIND, C. & ERNSTER, L. (1988). DT-Diaphorase-catalyzed two-electron reduction of various p-benzoquinone and 1,4-naphthoquinone epoxides. *Free Radical Biol. Med.*, **5**, 133.

BRYANT, D.W. & MCCALLA, D.R. (1980). Nitrofuran induced mutagenesis and error prone repair in *Escherichia coli. Chem-Biol Interactions*, **31**, 151.

BUSH, R.S., JENKIN, R.D.T., ALLT, W.E.C. & 4 others (1978). Definitive evidence for hypoxic cells influencing cure in cancer therapy. *Br. J. Cancer*, **37**, suppl. III, 302.

CHACON, E., MORROW, C.J., LEON, A.A., BORN, J.L. & SMITH, B.R. (1988). Regioselective formation of a misonidazole-glutathione conjugate as a function of pH during chemical reduction. *Biochem. Pharmacol.*, **37**, 361.

CHAPMAN, J.D. (1979). Hypoxic sensitizers: implications for radiation therapy. *N. Engl. J. Med.*, **301**, 1429.

CHAPMAN, J.D., FRANKO, A.J. & SHARPLIN, J. (1981). A marker for hypoxic cells in tumours with potential clinical applicability. *Br. J. Cancer*, **43**, 546.

COBBS, L.M., HACKER, T. & NOLAN, J. (1990). NAD(P)H nitroblue tetrazolium reductase levels in apparently normoxic tissues: a histochemical study correlating enzyme activity with binding of radiolabelled misonidazole. *Br. J. Cancer*, **61**, 524.

CREASEY, W.A. & THRALL, D.E. (1982). Pharmacokinetic and antitumour studies with the radiosensitizer misonidazole in dogs with spontaneous fibrosarcomas. *Am. J. Vet. Res.*, **43**, 1015.

FRANKO, A.J. (1986). Misonidazole and other hypoxia markers: metabolism and applications. *Int. J. Radiat. Oncol. Biol. Phys.*, **12**, 1195.

FRANKO, A.J. & KOCH, C.J. (1984). Binding of misonidazole to V79 spheroids and fragments of Dunning rat prostatic and human colon carcinomas *in vitro*. Diffusion of oxygen and reactive metabolites. *Int. J. Radiat. Oncol. Biol. Phys.*, **10**, 1333.

FRANKO, A.J., KOCH, C.J., GARRECHT, B.M., SHARPLIN, J. & HUGHES, D. (1987). Oxygen dependence of binding of misonidazole to rodent and human tumours *in vitro*. *Cancer Res.*, **47**, 5367.

FRANKO, A.J., RALEIGH, J.A., SUTHERLAND, R.G. & SODERLIND, K.J. (1989). Metabolic binding of misonidazole to mouse tissues. Comparison between labels on the ring and side chain, and the production of tritiated water. *Biochem. Pharmacol.*, **38**, 665.

FRANK. A.J. & SUTHERLAND, R.M. (1979). Oxygen diffusion distance and development of necrosis in multicell spheroids. *Radiat. Res.*, **79**, 439.

GARCIA, C.F., WEISS, L.M., LOWDER, J. & 4 others (1986). Quantitation and estimation of lymphocyte subsets in tissue sections. *Am. J. Clin. Pathol.*, **87**, 470.

GATENBY, R.A., KESSLER, H.B., ROSENBLUM, J.S. & 4 others (1988). Oxygen distribution in squamous cell carcinoma metastases and its relationship to outcome of radiation therapy. *Int. J. Radiat. Oncol. Biol. Phys.*, **14**, 831.

GIESE, A.C. (1968). *Cell Physiology*, 3rd edn. W.B. Saunders: Philadelphia.

GUYTON, A.C. (1979). *Physiology of the Human Body*. 5th edn. W.B. Saunders: Philadelphia.

HENDRY, J.H. (1979). Quantification of the radiotherapeutic importance of naturally hypoxic normal tissues from collated experiments with rodents using single doses. *Int. J. Radiat. Oncol. Biol. Phys.*, **5**, 971.

HIRST, D.G., DENEKAMP, J. & HOBSON, B. (1982). Proliferation of endothelial and tumour cells in three mouse mammary carcinomas. *Cell Tissue Kinet.*, **15**, 251.

JETTE, D.C., WIEBE, L.I. & CHAPMAN, J.D. (1983). Synthesis and *in vivo* studies of the radiosensitizer 4-(Br-82)-bromomisonidazole. *Int. J. Nucl. Med. Biol.*, **10**, 205.

KALLMAN, R.F. (1972). The phenomenon of reoxygenation and its implications for fractionated radiotherapy. *Radiology*, **105**, 135.

KENNEDY, K.A. (1987). Hypoxic cells as specific drug targets for chemotherapy. *Anti-Cancer Drug Design*, **2**, 181.

MAXWELL, R.J., WORKMAN, P. & GRIFFTHS, J. (1989). Demonstration of tumor selective retention of fluorinated nitroimidazole probes by F-19 magnetic resonance spectroscopy *in vivo*. *Int. J. Radiat. Oncol. Biol. Phys.*, **16**, 925.

MILLER, G.G., BEST, M.W., FRANKO, A.J., KOCH, C.J. & RALEIGH, J.A. (1989). Quantitation of hypoxia in multicellular spheroids by video image analysis. *Int. J. Radiat. Oncol. Biol. Phys.*, **16**, 949.

OWEN, L.N. & STEEL, G.G. (1969). The growth and cell population kinetics of spontaneous tumours in domestic animals. *Br. J. Cancer*, **23**, 493.

POPEL, A.S., PITMANN, R.N. & ELLSWORTH, M.L. (1989). Rate of oxygen loss from arterioles is an order of magnitude higher than expected. *Am. J. Physiol.*, **256**, H921.

RALEIGH, J.A., FRANKO, A.J., KOCH, C.J. & BORN, J.A. (1985). Binding of misonidazole to hypoxic cells in monolayer and spheroid culture. Evidence that a sidechain label is bound as efficiently as a ring label. *Br. J. Cancer*, **51**, 229.

RALEIGH, J.A., FRANKO, A.J., TREIBER, E.O., LUNT, J.A. & ALLEN, P.S. (1986). Covalent binding of a fluorinated 2-nitroimidazole to EMT/6 tumours in BALB/C mice. Detection by F-19 nuclear magnetic resonance at 2.3 T. *Int. J. Radiat. Oncol. Biol. Phys.*, **12**, 1249.

RALEIGH, J.A. & KOCH, C.J. (1990). The importance of thiols in the reductive binding of 2-nitroimidazoles to macromolecules. *Biochem. Pharmacol.* (in the press).

RALEIGH, J.A., MILLER, G.G., FRANKO, A.J., KOCH, C.J., FUCIARELLI, A.F. & KELLY, D.A. (1987). Fluorescence immunohistochemical detection of hypoxic cells in spheroids and tumours. *Br. J. Cancer*, **56**, 395.

RASEY, J.S., KOH, W., GRIERSON, J.R., GRUNBAUM, Z. & KROHN, K.A. (1989). Radiolabelled fluoromisonidazole as an imaging agent for tumour hypoxia. *Int. J. Radiat. Oncol. Biol. Phys.*, **17**, 985.

RAUTH, A.M., MCCLELLAND, R.A., MICHAELS, H.B. & BATTISTELLA, R. (1984). The oxygen dependence of the reduction of nitroimidazoles in a radiolytic model system. *Int. J. Radiat. Oncol. Biol. Phys.*, **10**, 1323.

SMITH, B.R. (1984). Hypoxia enhanced reduction and covalent binding of 2-(H-3)-misonidazole in the perfused rat liver. *Biochem. Pharmacol.*, **33**, 1379.

THOMLINSON, R.H. & GRAY, L.H. (1955). The histological structure of some human lung cancers and the possible implications for radiotherapy. *Br. J. Cancer*, **9**, 539.

TRACY, J.W., CATTO, B.A. & WEBSTER, L.T. (1983). Reductive metabolism of niridazole by adult *Schistosoma mansoni*. Correlation with covalent drug binding to parasite macromolecules. *Mol. Pharmacol.*, **24**, 291.

URTASUN, R.C., KOCH, C.J., FRANKO, A.J., RALEIGH, J.A. & CHAPMAN, J.D. (1986). A novel technique for measuring human tissue $Po2$ at the cellular level. *Br. J. Cancer*, **54**, 453.

VANHOUTTE, P.M. (1988). The endothelium – modulator of vascular smooth-muscle tone. *N. Engl. J. Med.*, **25**, 512.

VAN OS-CORBY, D.J., KOCH, C.J. & CHAPMAN, J.D. (1987). Is misonidazole binding to mouse tissues a measure of cellular $PO_2$? *Biochem. Pharmacol.*, **36**, 3487.

VARGHESE, A.J. (1983). Glutathione conjugates of misonidazole. *Biochem. Biophys. Res. Commun.*, **112**, 1013.

VARGHESE, A.J. & WHITMORE, G.F. (1980). Binding to cellular macromolecules as a possible mechanism for the cytotoxicity of misonidazole. *Cancer Res.*, **40**, 2165.

WHITE, R., WORKMAN, P. & OWEN, L. (1982). The pharmacokinetics in mice and dogs of nitroimidazole radiosensitizers and chemosensitizers more lipophilic than misonidazole. *Int. J. Radiat. Oncol. Biol. Phys.*, **8**, 473.

WORDINGER, R.J., MILLER, G.W. & NICODENUS, D.S. (1987). *Manual of Immunoperoxidase Techniques*. ASCP Press: Chicago.